# Clarithromycin attenuates IL-13–induced periostin production in human lung fibroblasts

**DOI:** 10.1186/s12931-017-0519-8

**Published:** 2017-02-20

**Authors:** Kosaku Komiya, Shoichiro Ohta, Kazuhiko Arima, Masahiro Ogawa, Shoichi Suzuki, Yasutaka Mitamura, Satoshi Nunomura, Yasuhiro Nanri, Tomohito Yoshihara, Atsushi Kawaguchi, Jun-ichi Kadota, Bruce K. Rubin, Kenji Izuhara

**Affiliations:** 1 Division of Medical Biochemistry, Department of Biomolecular Sciences, Saga Medical School, 5-1-1 Nabeshima, Saga, 849-8501 Japan; 2 Department of Pediatrics, Virginia Commonwealth University School of Medicine, 1217 East Marshall Street, Bldg: KMSB I, Room: 215, Richmond, Virginia 23298 USA; 3 Respiratory Medicine and Infectious Diseases, Oita University Faculty of Medicine, 1-1 Idaigaoka Hasama, Yufu, 879-5503 Japan; 4 Clinical Research Center of Respiratory Medicine, Tenshindo Hetsugi Hospital, 5956 Nihongi Nakahetsugi, Oita, 879-7761 Japan; 5 Asia International Institute of Infectious Disease Control, Teikyo University, 2-11-1 Kaga Itabashi-ku, Tokyo, 173-8605 Japan; 6 Center for Comprehensive Community Medicine, Saga Medical School, 5-1-1 Nabeshima, Saga, 849-8501 Japan

**Keywords:** Macrolide, Periostin, Asthma, Fibroblast, IL-13

## Abstract

**Background:**

Periostin is a biomarker indicating the presence of type 2 inflammation and submucosal fibrosis; serum periostin levels have been associated with asthma severity. Macrolides have immunomodulatory effects and are considered a potential therapy for patients with severe asthma. Therefore, we investigated whether macrolides can also modulate pulmonary periostin production.

**Methods:**

Using quantitative PCR and ELISA, we measured periostin production in human lung fibroblasts stimulated by interleukin-13 (IL-13) in the presence of two 14-member–ring macrolides—clarithromycin or erythromycin—or a 16-member–ring macrolide, josamycin. Phosphorylation of signal transducers and activators of transcription 6 (STAT6), downstream of IL-13 signaling, was evaluated by Western blotting. Changes in global gene expression profile induced by IL-13 and/or clarithromycin were assessed by DNA microarray analysis.

**Results:**

Clarithromycin and erythromycin, but not josamycin, inhibited IL-13–stimulated periostin production. The inhibitory effects of clarithromycin were stronger than those of erythromycin. Clarithromycin significantly attenuated STAT6 phosphorylation induced by IL-13. Global gene expression analyses demonstrated that IL-13 increased mRNA expression of 454 genes more than 4-fold, while decreasing its expression in 390 of these genes (85.9%), mainly “extracellular,” “plasma membrane,” or “defense response” genes. On the other hand, clarithromycin suppressed 9.8% of the genes in the absence of IL-13. Clarithromycin primarily attenuated the gene expression of extracellular matrix protein, including periostin, especially after IL-13.

**Conclusions:**

Clarithromycin suppressed IL-13–induced periostin production in human lung fibroblasts, in part by inhibiting STAT6 phosphorylation. This suggests a novel mechanism of the immunomodulatory effect of clarithromycin in asthmatic airway inflammation and fibrosis.

**Electronic supplementary material:**

The online version of this article (doi:10.1186/s12931-017-0519-8) contains supplementary material, which is available to authorized users.

## Background

The immunomodulatory effects of macrolides were first described in patients with diffuse panbronchiolitis in 1998 [1]. Macrolide immunomodulation was found to be independent of antibiotic properties [2]. Their effects include modulation (both increasing and decreasing) of inflammatory cytokine production, decreasing airway mucus hypersecretion, and blocking bacterial biofilm formation and virulence factor production [2–5]. Macrolide therapy has been recommended for chronic obstructive pulmonary disease, cystic fibrosis, non-cystic fibrosis bronchiectasis, and severe asthma [6–10]. In patients with asthma, long-term macrolide therapy was reported to improve airflow, quality of life, and airway hypersensitiveness [11].

Periostin is an extracellular matrix protein that is associated with eosinophilic airway inflammation and the severity of asthma. Periostin may enhance type 2 inflammation and mucus hypersecretion [12–15]. Periostin is reported to be the most robust biomarker predicting the effectiveness of lebrikizumab, an anti-IL-13 antibody, for treating asthma [16–18]. As macrolides also affect type 2-dominated inflammation in asthma, we hypothesized that macrolide therapy may attenuate IL-13 stimulated periostin production and inflammatory gene expression in human lung fibroblasts.

## Methods

### Cell culture

MRC5 cells, a human embryonic lung fibroblast cell line (Riken BioResource Center, Tsukuba, Japan), were cultured with Dulbecco modified Eagle medium (Sigma-Aldrich, St. Louis, MO, USA) supplemented with 10% fetal calf serum, 100 μg/mL streptomycin, and 100 U/mL penicillin G. MRC5 cells (7 × 10^4^ cells per well) were placed in 24-well plates (Nunc, Roskilde, Denmark) and cultured in 5% CO_2_ humidified atmosphere at 37 °C with or without clarithromycin, erythromycin (Wako Pure Chemical Industries, Osaka, Japan), josamycin (Sigma-Aldrich), ampicillin (Sigma-Aldrich), or dexamethasone (Wako Pure Chemical Industries). Clarithromycin was kindly supplied by Taisho Toyama Co., Ltd. (Tokyo, Japan). Clarithromycin, erythromycin, josamycin, and ampicillin were dissolved in ethanol (EtOH, Wako) to therapeutic concentrations [19, 20]. Dexamethasone was dissolved in EtOH to 100 nM [21]. The final concentration of EtOH added to cells was 0.5%. After 24 h of culture, cells were stimulated by 50 ng/mL human recombinant IL-13 (Peprotech, Rocky Hill, NJ, USA) for 24 h. Cell viability was evaluated using WST-8 assay (Cell Count Reagent SF, Nacalai Tesque, Kyoto, Japan).

### Real-time PCR

Total RNA was extracted using RNAiso Plus (Takara Bio, Otsu, Japan), and reverse-transcribed with ReverTra Ace (Toyobo, Osaka, Japan). Quantitative PCR reactions were performed with cDNA on a StepOnePlus real-time PCR System (Life Technologies, Carlsbad, CA, USA) using the Thunderbird SYBR qPCR mix (Toyobo). PCR primers were as follows: periostin, forward primer, 5’-CTGCCAAACAAGTTATTGAGCTGGC-3’, reverse primer, 5’-AATAATGTCCAGTCTCCAGGTTG-3’ and glyceraldehyde-3-phosphate dehydrogenase (GAPDH), forward primer, 5’-TCACCACCATGGAGAAGGC-3’, reverse primer, 5’-GCTAAGCAGTTGGTGGTGCA-3’. Threshold cycles of primer probes were normalized by GAPDH Additional file 1.

### ELISA

Periostin ELISA was performed using Periostin ELISA Kit® (Shino-Test Corp., Tokyo, Japan) according to the manufacturer's instruction.

### Western blot analysis

Western blotting for STAT6 and phosphorylated STAT6 was performed as previously described [22]. MRC5 cells were stimulated with the indicated concentration of IL-13 at 37 °C for 1, 3, or 6 h. The cell lysates were applied to SDS-PAGE and then electrophoretically transferred to polyvinylidene difluoride membranes. Membranes were incubated with either anti-phosphotyrosyl STAT6 antibody (Cell Signaling Technology, Beverly, MA, USA) or anti-STAT6 Ab (Santa Cruz Biotechnology, Santa Cruz, CA, USA), followed by incubation with secondary Abs conjugated to horseradish peroxidase. The signals were visualized with an enhanced chemiluminescence system (Thermo Scientific, Waltham, MA, USA) and LAS-3000 (GE Healthcare, Pittsburg, PA, USA).

### DNA microarray analysis

MRC5 cells were stimulated with 50 ng/mL IL-13 for 24 h in the presence or absence of 5.0 × 10^−5^ M clarithromycin to evaluate not only the primary gene transcription but also the secondary gene expression caused by the primary products. Total RNA with an RNA integrity number more than 7.0 was applied to Agilent Expression Array (SurePrint G3 Human GE8x60K v2 Microarray, Takara Bio). The calculated relative signal intensity values were presented on a heat map and subjected to MultiExperiment Viewer (MeV) v4.9 software (Dana-Farber Cancer Institute, Boston, MA, USA). For gene ontology analysis, the Database for Annotation Visualization and Integrated Discovery (DAVID) tool (National Cancer Institute, Frederick, MA, USA) was used. This database includes the Gene Ontology Database (http://geneontology.org/).

### Statistical analysis

Statistical analyses were performed using the Prism 5.0 software (GraphPad Software, La Jolla, CA, USA) and the IBM SPSS statistics 21.0 software package (IBM SPSS, Tokyo, Japan). Data were presented as mean ± SD. The significance of differences was assessed using an unpaired Student’s *t*-test, except for the multiple comparisons of compounds, which were done using the ANOVA plus post-test (Tukey). *P* values less than 0.05 were considered statistically significant.

## Results

### Clarithromycin inhibits periostin production in MRC5 cells

**Fig. 1:**
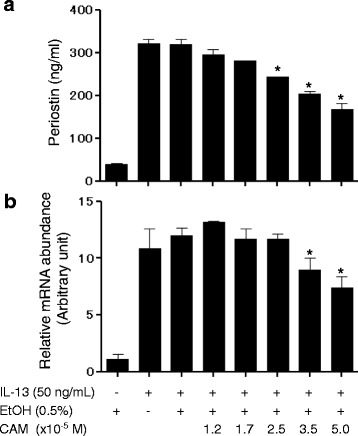
Effects of clarithromycin on periostin production in MRC5 cells. Periostin protein in supernatant measured by ELISA (**a**) or mRNA by qPCR (**b**). Bars are depicted as mean ± SD. The same experiments were performed twice for (A) and three times for (B). A representative result of three individual experiments is shown. *; *P* < 0.05 compared with IL-13 (50 ng/mL) plus EtOH (0.5%)

We first examined whether clarithromycin affects IL-13–stimulated periostin production. We chose the concentrations of clarithromycin based on an earlier reference showing the clarithromycin concentration in epithelial lining fluid after taking clarithromycin [19]. IL-13 increased periostin expression approximately 10-fold compared to control as reported previously [22] (Fig. 1). Clarithromycin significantly attenuated IL-13 stimulated periostin in a dose-dependent manner from 318 ± 19 ng/mL with no clarithromycin to 168 ± 18 ng/mL (at 5.0 × 10^−5^ M, *P* < 0.001). Cellular viability was not affected by clarithromycin at these concentrations (data not shown). These results suggest that clarithromycin inhibits periostin production in a dose-dependent manner in human fibroblasts.

### Effects of macrolides, dexamethasone, and ampicillin on periostin production

**Fig. 2:**
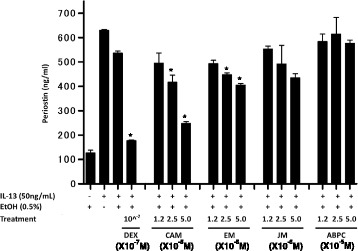
Effects of erythromycin, josamycin, and ampicillin on periostin production in MRC5 cells. MRC5 cells were cultured for 24 h in the presence of the indicated concentrations of dexamethasone, clarithromycin, erythromycin, josamycin, or ampicillin. Then the cells were stimulated with 50 ng/mL IL-13 for 24 h. Periostin protein in supernatant was measured by ELISA. The same experiments were performed twice. Bars are depicted as mean ± SD. A representative result of three individual experiments is shown. *; *P* < 0.05 compared with IL-13 (50 ng/mL) plus EtOH (0.5%)

The immunomodulatory effects of macrolides have been reported to be associated with the size of the macrolactam ring; macrolides with 14- or 15-member rings exhibit immunomodulatory properties, while these properties are absent or attenuated in the 16-member–ring macrolide antibiotics [2]. Clarithromycin, like erythromycin, is a 14-member–ring macrolide, while josamycin, has a 16-member ring. Clarithromycin robustly inhibited periostin production at both 2.5 × 10^−5^ M (*P* < 0.01) and 5.0 × 10^−5^ M (*P* < 0.001) (Fig. 2). Erythromycin inhibited IL-13–stimulated periostin production more weakly than clarithromycin but significantly at 5.0 × 10^−5^ M (*P* < 0.01). However, josamycin had no effect on periostin production (*P* = 0.3020), nor did ampicillin (*P =* 0.6052). Dexamethasone, at a concentration of 10^−7^ M, also attenuated periostin production (*P* < 0.001). These results suggest that clarithromycin and erythromycin, both having 14-member rings, but not josamycin, with 16-member ring, inhibit periostin production induced by IL-13 in human lung fibroblasts.

### Clarithromycin inhibits IL-13–induced STAT6 phosphorylation

**Fig. 3:**
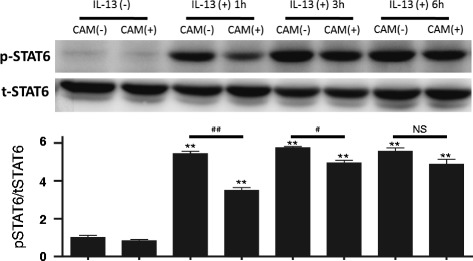
Clarithromycin inhibits IL-13–stimulated STAT6 phosphorylation. MRC5 cells were stimulated by exposure to 50 ng/mL IL-13 in the presence or absence of clarithromycin. Phosphorylated STAT6 in the cell lysates of MRC5 cells was analyzed by Western blot. Bars are depicted as mean ± SD. The same experiments were performed three times; a representative result of three individual experiments is shown. **; *P* < 0.001 compared with control, ^#^; *P* < 0.05, ^##^; *P* < 0.001 compared with no clarithromycin, NS; not significant

IL-13 receptor activation signals through STAT6 phosphorylation [23]. Tanabe et al. reported that clarithromycin inhibits STAT6 phosphorylation in human bronchial epithelial cells [20]. We hypothesized that STAT6 inhibition by clarithromycin would decrease IL-13**–**stimulated periostin expression in MRC5 fibroblasts. IL-13 induced STAT6 phosphorylation within 1 h, and this continued for more than 6 h (Fig. 3). Clarithromycin partially attenuated STAT6 phosphorylation just 1 h after IL-13 exposure. These results suggest that clarithromycin attenuates periostin production induced by IL-13 at least partially by inhibiting STAT6 phosphorylation.

### IL-13 and clarithromycin affect gene expression in MRC5 cells

**Fig. 4:**
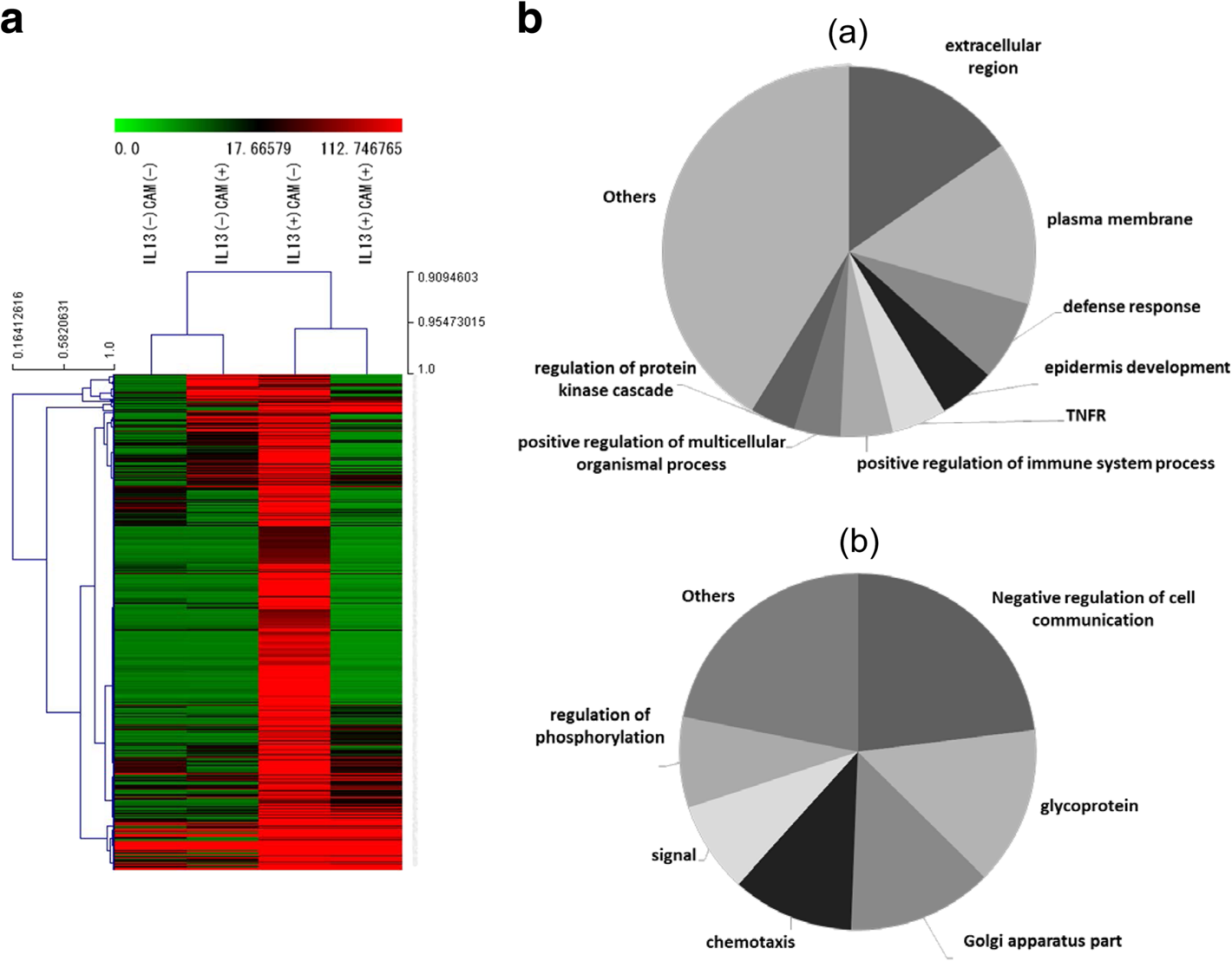
Global gene expression analysis. **a** A hierarchical clustering using DNA microarray analysis of IL-13– and clarithromycin-dependent gene expression changes in MRC5 cells. Genes whose expression were increased more than 4-fold by IL-13 without clarithromycin are displayed. The *rows* represent genes; the experimental conditions are shown as columns. The color represents the expression level of the gene (*Red* represents high expression, while green represents low expression). The dendrograms provide some qualitative means of assessing the similarity between genes and between experimental conditions. Among IL-13–inducible genes, expression of 390 genes was decreased by 50% or more, 56 genes were unaffected (0.5 to 1.5-fold), and expression of 8 genes was further increased by clarithromycin. **b** GO term decreased (*a*) or unchanged (*b*) by clarithromycin among genes increased 4-fold by IL-13. Gene enrichment in each GO term was analyzed by Fisher’s exact test. *P* values for (*a*) are extracellular region, 6.9E-04; plasma membrane, 1.6E-04; defense response, 6.1E-04; epidermis development, 1.3E-03; TNFR, 2.3E-02; positive regulation of immune system process, 4.5E-03; positive regulation of multicellular organismal process; 5.1E-03; and regulation of protein kinase cascade, 5.6E-03. *P* values for (*b*) are negative regulation of cell communication, 9.4E-03; glycoprotein, 5.6E-03; Golgi apparatus part, 2.1E-02; chemotaxis, 4.0E-02; signal, 7.0E-02; and regulation of phosphorylation, 2.4E-01

To examine the selectivity of the inhibitory effects by clarithromycin on IL-13–induced expression, we analyzed the changes in IL-13– and clarithromycin-dependent gene expression. Global gene expression analysis showed that IL-13 increased mRNA expression of 454 genes more than 4-fold. Of these 454 genes, clarithromycin exposure attenuated expression of 390 (85.9%, Fig. 4a). The Gene Ontology (GO) terminology provides uniform and consistent descriptions of genes and gene products (http://geneontology.org/). We categorized the genes affected by IL-13 and clarithromycin using GO terminology. IL-13 primarily increased mRNA in “extracellular,” “plasma membrane,” or “defense response” genes, whereas clarithromycin suppressed these categories of genes, but had no effect on “negative regulation of cell communication,” “glycoprotein,” or “Golgi apparatus” genes (Fig. 4b and Table 1). In GO terminology, periostin belongs to an extracellular region gene. In the absence of IL-13 stimulation, clarithromycin suppressed 5758 of 58718 genes (9.8%). The genes categorized as “extracellular region,” “plasma membrane,” or “defense response,” which were suppressed by clarithromycin in the presence of IL-13, were only partially attenuated in the absence of IL-13: 9 of 56 genes (16.1%), 8 of 93 genes (8.6%), and 2 of 16 genes (12.5%), respectively. These findings suggest that clarithromycin inhibits mainly “extracellular,” “plasma membrane,” or “defense response” genes induced by IL-13 in which periostin is involved.

**Table 1:** Representative genes that were increased more than 4-fold by IL-13 according to the global gene expression analysis (see Figure 4.)

(A) Cluster	Gene Symbol	CAM effect
Extracellular	PLXDC1	0.0342
Extracellular	SLIT2	0.4594
Extracellular	POSTN	0.4979
Extracellular	WNT10B	0.4239
Plasma membrane	CACNA1D	0.0807
Plasma membrane	RTP1	0.1379
Plasma membrane	SYT8	0.2078
Plasma membrane	RALGPS1	0.2754
Plasma membrane	APC	0.2138
Defense response	CD40	0.0131
Defense response	NOS2	0.3132
Defense response	CD19	0.1667
Defense response	IFNL1	0.0426
Defense response	CXCL1	0.2197
(B) Cluster	Gene Symbol	CAM effect
Glycoprotein	IL17RA	0.9564
Glycoprotein	ST8SIA1	0.6773
Glycoprotein	SPINT2	0.8788
Glycoprotein	IL1RL1	0.7990
Glycoprotein	SULF1	0.5449
Glycoprotein	APLNR	1.0210
Glycoprotein	HS3ST1	0.9854
Chemotaxis	CCL26	1.2418
Chemotaxis	IL6	0.8210
Chemotaxis	PTGDR2	0.7217
Signal regulator	SOCS1	1.1385
Signal regulator	CISH	0.8930
(C) Cluster	Gene Symbol	IL-13 effect
Chemotaxis	CCL26	35.6732
Glycoprotein	HS3ST1	12.2410
Glycoprotein	ST8SIA1	10.7933
Chemotaxis	IL6	10.1221
Signal regulator	CISH	8.9751
Chemotaxis	PTGDR2	7.2666
Unassigned	LINC00971	7.2156
Signal regulator	SOCS1	6.3270
Glycoprotein	IL17RA	5.9221
Extracellular	SLIT2	5.7987
(D) Cluster	Gene Symbol	CAM effect
Plasma membrane	SLC22A20	0.1237
Unassigned	XLOC_006850	0.1403
Unassigned	XLOC_014512	0.1420
Unassigned	SPDYE8P	0.1491
Defense response	CD19	0.1667
Unassigned	LOC100129675	0.1696
Unassigned	LOC102724783	0.1880
Plasma membrane	SYT8	0.2078
Centriole	SASS6	0.2079
Plasma membrane	APC	0.2138

(A) Genes suppressed by 50% or more by clarithromycin. (B) Genes not affected by clarithromycin. The clarithromycin (CAM) effect denotes the ratio of signal intensities obtained by clarithromycin treatment compared with no treatment. (C) The ten top-ranked ten genes induced by IL-13. (D) The ten top-ranked genes downregulated by clarithromycin in the presence of IL-13. The clarithromycin (CAM) effect denotes the ratio of signal intensities obtained by clarithromycin treatment compared with no treatment. The IL-13 effect denotes the ratio of signal intensities obtained by IL-13 stimulation compared with no stimulation

## Discussion

It has been consistently reported that macrolides with 14- and 15-member rings have much greater immunomodulatory effects than the 16-member ring macrolides [2]. In this study, we showed that clarithromycin, a 14-member–ring macrolide, showed the strongest inhibitory effects on periostin expression induced by IL-13 among the examined macrolides. Erythromycin, another 14-member–ring macrolide, showed fewer inhibitory effects while josamycin, a 16-member–ring macrolide, had no such effects. The details of what causes these differences in the inhibitory effects are thus far unclear; however, our results are consistent with these reports in that clarithromycin had the greatest suppressive effect on IL-13–induced periostin expression [2].

The Janus kinase (JAK)-STAT6 pathway is key to IL-13 signaling [24]. We confirmed that clarithromycin suppressed the phosphorylation of STAT6, but the ability of clarithromycin to attenuate periostin production may not entirely be explained by inhibiting STAT6 phosphorylation. There are several reports that STAT6 inhibition stops most periostin expression in lung fibroblasts [25]. These data indicate that STAT6 is the exclusive regulator of periostin expression in lung fibroblasts. On the other hand, we have recently demonstrated that the periostin level is decreased in bronchial epithelial cells by inhibitors against extracellular signal-regulated kinase (ERK) and nuclear factor-kappa B (NF-κB) in addition to STAT6, suggesting that in other cell types, the ERK and NF-κB pathways are involved in periostin production [26]. Additionally, it has been reported that the ERK signaling pathway positively regulates JAK1/STAT6 activity in T cells [27]. On the other hand, macrolides are known to decrease ERK and NF-κB signaling pathways [28]. Tanabe et al. reported that clarithromycin attenuates these pathways and the JAK-STAT6 pathway [20]. Taken together, these results suggest that macrolides may attenuate periostin production via the ERK or NF-κB signaling pathways in addition to the JAK/STAT6 pathway. Clarithromycin may affect STAT6 signaling by downregulating IL-13Rα1/IL-4Rα. We performed flow cytometry to investigate the surface expression of the cytokine receptors on MRC5 cells upon treatment with clarithromycin (Additional file 2: Figure S1A). The expression of IL-13Rα1 was not affected by clarithromycin. Although a statistically significant decrease of IL-4Rα expression was observed, it seemed too slight to explain the considerable attenuation of the periostin production by clarithromycin. To confirm the expression of these receptors at the transcriptional level, we also performed quantitative PCR, finding no suppressive effect by clarithromycin (Additional file 2: Figure S1B). Consequently, we conclude that the inhibition of the STAT6 signaling by clarithromycin is not mainly due to downregulation of the IL-13Rα1/IL-4Rα expression.

We found that clarithromycin showed significantly suppressive effects on IL-13–inducble genes (Fig. 4). These specific genes, whose expression was attenuated by clarithromycin after IL-13 exposure, were dominantly categorized as “extracellular region,” “plasma membrane,” and “defense response” genes, among which asthma-related CD40, NOS2, and CXCL1 (Table 1A) were included. The improvement of asthma symptoms by clarithromycin may be attributed to the downregulation of these genes in addition to periostin. In contrast to suppression of genes activated by IL-13, constitutive expression of these genes was less affected by clarithromycin. Macrolides are classified as ‘immunomodulators’ and decrease hyperinflammation without impairing the normal immune system against infection, as differentiated from immunosuppressive agents such as glucocorticosteroids [2]. The detailed mechanism of how macrolides select for suppressive genes still remains unclear; however, our present finding that clarithromycin selectively suppresses IL-13–inducible genes including periostin may shed light on this mechanism. Extracellular matrix proteins constitute a positive feedback loop in lung fibrosis [23, 29]. Masuoka et al. showed that type 2 cytokines stimulated fibroblasts to produce periostin, interacting with αv integrin, a functional periostin receptor, on keratinocytes [23]. Inhibition of periostin or αv integrin prevented the development or progression of allergen-induced skin inflammations, including fibrosis. Macrolides are reported to have anti-fibrotic effects [30], implying that they may attenuate fibrosis by modulating extracellular matrix proteins. Serum periostin levels are significantly increased in asthmatic patients [12]. The role of periostin on fibrogenesis has been explored, showing that epithelial cell-derived periostin increased secretion of type 1 collagen from airway fibroblasts [14]. Attenuation of periostin production by macrolides may decrease both asthmatic airway inflammation and fibrosis.

This study has a certain limitation. We selected the concentration of clarithromycin (5.0 × 10^−5^ M) based on a previous report showing the clarithromycin concentration in epithelial lining fluid after taking clarithromycin [19]. Our study and most of the studies assessing the effects of macrolides used the unified concentrations for each drug when comparing the immunomodulatory effects among macrolides with different types of rings [4, 5]. We did not evaluate whether these drugs at the same concentrations were equally efficacious with other assay such as bactericidal activity. Thus, the results do not necessarily prove actual intrinsic differences in the inhibitory efficacy of these drugs.

## Conclusions

Clarithromycin suppressed IL-13–induced periostin production in human lung fibroblasts, in part through inhibition of STAT6 phosphorylation. This suggests a novel mechanism of the immunomodulatory effect of clarithromycin in asthmatic airway inflammation and fibrosis.

## Additional files

Additional file 1: Figure S1. Expression of IL-4Rα and IL-13Rα1 in MRC5 cells. (A) Cell surface expression of IL-4Rα and IL-13Rα1 was assessed by flow cytometry. Mean fluorescent intensities (MFI) of the stained cells are shown. (B) Expression of mRNA of the indicated genes was assessed by quantitative RT-PCR. Fold changes over vehicle are shown. Black columns, clarithromycin (CAM); gray columns, vehicle. Statistical analyses were performed using Bonferroni’s multiple comparison test. P values of 0.05 or less were regarded significant. NS, not significant; MFI, mean fluorescent intensity. (PPT 121 kb)
Additional file 2: Materials and Methods. (DOCX 21 kb)

## Abbreviations

CAM: Clarithromycin
CD40: Cluster of differentiation antigen 40
CXCL1: C-X-C motif chemokine ligand 1
ELISA: Enzyme-linked immunosorbent assay
ERK: Extracellular signal-regulated kinase
EtOH: Ethanol
GAPDH: Glyceraldehyde-3-phosphate dehydrogenase
GO: Gene ontology
IL-13: Interleukin-13
IL-13α1: Interleukin-13 receptor α1
IL-4: Interleukin-4
IL-4α: Interleukin-4 receptor α
JAK: Janus kinase
NF-κB: Nuclear factor-kappa B
NOS2: Nitric oxide synthase 2
PCR: Polymerase chain reaction
qPCR: Quantitative PCR
STAT6: Signal transducer and activator of transcription 6
WST-8: Water-soluble tetrazolium salt 8
