# Supplementary figures and images for: Clarithromycin attenuates IL-13–induced periostin production in human lung fibroblasts
Source: Respir Res. 2017 Feb 20;18:37. doi: 10.1186/s12931-017-0519-8 (PMC5319114; doi:10.1186/s12931-017-0519-8)

## Slide 1
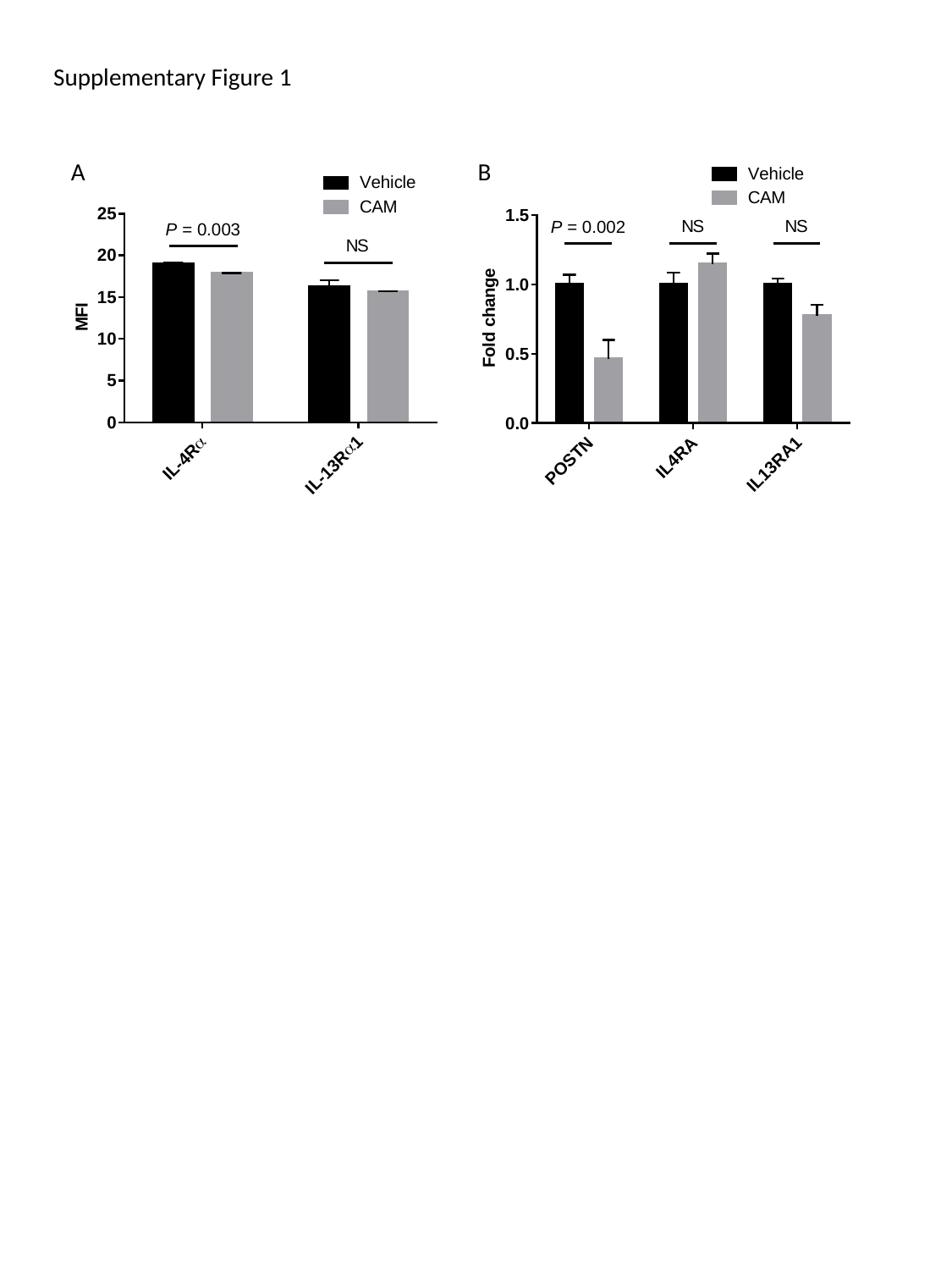

Supplementary Figure 1
A
B

Supplement: Additional file 1: Figure S1. — Expression of IL-4Rα and IL-13Rα1 in MRC5 cells. (A) Cell surface expression of IL-4Rα and IL-13Rα1 was assessed by flow cytometry. Mean fluorescent intensities (MFI) of the stained cells are shown. (B) Expression of mRNA of the indicated genes was assessed by quantitative RT-PCR. Fold changes over vehicle are shown. Black columns, clarithromycin (CAM); gray columns, vehicle. Statistical analyses were performed using Bonferroni’s multiple comparison test. P values of 0.05 or less were regarded significant. NS, not significant; MFI, mean fluorescent intensity. (PPT 121 kb) [file 12931_2017_519_MOESM1_ESM.ppt]
