# Supplementary material for: Clarithromycin attenuates IL-13–induced periostin production in human lung fibroblasts
Source: Respir Res. 2017 Feb 20;18:37. doi: 10.1186/s12931-017-0519-8 (PMC5319114; doi:10.1186/s12931-017-0519-8)
Supplement: Additional file 2: — Materials and Methods. (DOCX 21 kb) [file 12931_2017_519_MOESM2_ESM.docx]

**SUPPLEMENTARY MATERIALS AND METHODS**

**Flow cytometry**

A monoclonal antibody for IL-4Rα (clone 25463) was purchased from Millipore, Birrelica, MA. A monoclonal antibody for IL-13Rα1 (clone SS12B) was established in house. Mice were immunized with drosophila silkworm-derived recombinant extracellular domain of human IL-13Rα1 and splenocytes from the mice were fused with Sp2/O myeloma cells using a standard protocol with polyethylene glycol. MRC5 cells treated with clarithromycin or vehicle (0.5% ethanol) for 48 hours were stained by the primary antibodies, followed by BB515-probed goat anti-mouse Ig antibody (BD Biosciences, Franklin Lakes, NJ). Flow cytometric analyses were conducted using FACSCalibur cytometer (BD Biosciences) and WinMDI software (The Scripps Research Institute, La Jolla, CA).

**Additional PCR primers**

IL-4Rα, forward primer, 5’- ATCAGATTCCCAACCCAGCC -3’, reverse primer, 5’-GGCAGCCTTGTGAGGATCTT-3’; IL-13Rα1, forward primer, 5’-GGTCCCTGGTGTTCTTCCTG-3’, reverse primer, 5’-TGCGACGATGACTGGAACAA-3’

**SUPPLEMENTARY FIGURE LEGENDS**

**Supplementary Figure 1**. Expression of IL-4Rα and IL-13Rα1 in MRC5 cells. (A) Cell surface expression of IL-4Rα and IL-13Rα1 was assessed by flow cytometry. Mean fluorescent intensities (MFI) of the stained cells are shown. (B) Expression of mRNA of the indicated genes was assessed by quantitative RT-PCR. Fold changes over vehicle are shown. Black columns, clarithromycin (CAM); gray columns, vehicle. Statistical analyses were performed using Bonferroni’s multiple comparison test. *P* values of 0.05 or less were regarded significant. NS, not significant; MFI, mean fluorescent intensity.
